# Auxin! here you go again: Spatiotemporal dynamic regulation of auxin promotes proper nodule formation in *Medicago truncatula*

**DOI:** 10.1093/plphys/kiaf177

**Published:** 2025-05-02

**Authors:** Gunjan Sharma, Héctor H Torres-Martínez

**Affiliations:** Assistant Features Editor, Plant Physiology, American Society of Plant Biologists; School of Biosciences, University of Birmingham, Edgbaston B15 2TT, UK; Assistant Features Editor, Plant Physiology, American Society of Plant Biologists; Department of Biology, Stanford University, Stanford, CA 94305, USA

Legumes such as peas, pulses, and soybeans are a primary source of proteins. Legumes have a unique property to produce knob-like structures called nodules on their roots after infection by nitrogen-fixing bacteria, rhizobia. Legumes and rhizobia form a mutual symbiotic relationship in which plants provide nutrients and rhizobia provide nitrates to plants by fixing atmospheric nitrogen (Soltis et al. 1995). The symbiotic relationship between legumes and rhizobia is crucial and beneficial to reduce the use of nitrogen fertilizers (Goyal et al. 2021). Therefore, understanding the process of symbiosis and nodule formation in different legume species is of great interest.

Nodulation requires a sophisticated array of coordinated cellular processes in specific root cells involving an interplay of plant hormones (Lin et al. 2020; Liu et al. 2023). A pioneering study reported that the auxin influx transporter proteins AUXIN RESISTANT 1 (AUX1) and LIKE-AUX1 (LAX) are expressed in nodule primordia (de Billy et al. 2001). Auxins are known to essentially regulate all plant developmental pathways (Teale et al. 2006). It is no surprise that auxin promotes root nodule development through cell cycle control and vascular tissue specialization for transporting water and nutrients (Kohlen et al. 2018; Lin et al. 2020).

A growing body of evidence has further strengthened the idea that differential auxin distribution is necessary to induce nodule development. However, how the auxin responsive gene network shapes the formation and maintenance of precise auxin concentrations is still not fully understood.

In a recently published article in *Plant Physiology*, Xiao et al. (2025) have dissected the expression pattern of auxin biosynthesis, *YUCCA* (*YUC*), and transport *PIN-formed* (*PIN*) genes during nodule primordium initiation and development. Authors revealed that auxin is present even before the start of cell division in pericycle cells and its concentration is dynamically maintained in subsequent stages of root nodule primordium organogenesis. Interestingly, auxin is mainly synthesized at the site of nodule primordia development instead of long-distance transport from shoots to roots, known as acropetal transport.

To investigate the auxin gradient during nodule primordium initiation and development, the authors introduced an artificial auxin-responsive *DR5-GUS* reporter into *Medicago truncatula* plants as a proxy for observing the auxin concentration gradient (Ulmasov et al. 1997). Medicago plants expressing *DR5-GUS* reporter were subjected to infection with rhizobia, and the formation of nodule primordia and auxin gradients was observed from 12 to 96 h post infection. Progression of nodule primordium development was divided into progressive stages from 0 to VI based on the initiation of cell divisions in pericycle, cortical, and nodule meristem cells. Interestingly, DR5-GUS was expressed in stage 0 in 6 to 8 pericycle cells before the onset of divisions (Figure). The auxin reporter was visible in subsequent stages (I to VI) in dividing pericycle, endodermis, cortex, nodule primordium, vasculature, and nodule meristem. However, DR5-GUS expression was dramatically reduced in inner cortical derived layers during later stages, and outer cortical derived layers exhibited auxin response maxima potentially for nodule meristem formation (Figure).

**Figure.**
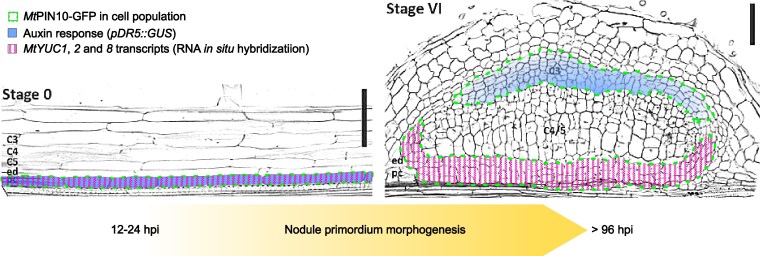
Schematic representation of auxin distribution mechanism during *Medicago truncatula* nodule primordium development. In this research, Xiao et al. (2025) expanded our understanding of how the auxin synthesis and distribution mechanisms play a critical role in proper nodule development. The authors showed that the auxin accumulation in pericycle cells at nodule primordium development stage 0 is primarily driven by local auxin biosynthesis mediated by *MtYUC1*, *2*, and *8*. *MtYUC1*, *2*, and *8* expression remains restricted at pericycle and endodermis derivatives throughout morphogenesis. On the other hand, *MtPIN-FORMED 2*, *4*, and *10*—auxin efflux carriers—exhibit a differential and dynamic spatiotemporal expression pattern. Notably, subcellular localization of MtPIN10 aligns with auxin response pattern during nodule primordium development. The schematic was created using Inkscape. Cell-outlined nodule primordia were adapted from the highlighted article (Xiao et al. 2025), Fig. 1 (stage 0 and VI), and processed in ImageJ to get binary images. Scale bars = 75 *µ*m.

To further explore the auxin distribution in nodule primordium development, the authors employed the in situ hybridization technique to observe the expression of known bona fide auxin biosynthetic *YUC* genes in fixed Medicago roots that have been inoculated with rhizobia. Expression of the *YUC* genes coincided with *DR5-GUS* reporter in rhizobia-inoculated roots during nodule stage 0 in pericycle cells and in developing nodule vasculature during later stages (Figure). Surprisingly, these *YUC* genes were not expressed in cortical cell layers in contrast to the observed *DR5* expression.

To address the potential auxin transport from pericycle to cortical cell layers, Xiao et al. (2025) studied the expression of Medicago auxin export carrier *PINs* and influx carrier *LAX2* using in situ hybridization approach. Authors revealed nodule primordia stage–specific expression patterns of *MtPINs* and *MtLAX2* facilitating the formation of auxin maxima in pericycle and cortical cell derivatives throughout nodule organogenesis. The authors' claim of auxin transport from pericycle to cortical cells was further strengthened by subcellular localization of GFP-tagged MtPIN10 transporter protein in stages II to IV of nodule primordium development. In later stages (V to VI), *MtPIN10* expression was predominant in cortex derivatives associated with nodule meristem (Figure).

To further investigate the role of YUCs and PIN proteins in precise auxin distribution, authors performed a combinatorial approach using RNA interference and YUC inhibitor 4-phenoxyphenylboronic acid to reduce the *MtYUCs* and *MtPIN* expression/activity. The treatment of roots with 4-phenoxyphenylboronic acid before rhizobia infection severely reduced the root nodule formation. A simultaneous downregulation of *MtYUC* members in constitutive and pericycle-specific manner reduced root nodule formation between 80% and 60%. Similarly, Medicago *PINi* (an *RNAi* construct to reduce *MtPIN2*, *4*, *and 10* expressions simultaneously) plants exhibited significantly reduced nodule numbers. Interestingly, pericycle- and symbiosis-specific downregulation of *PIN*s substantially reduced nodule number along with poorly developed nodule meristem. These observations establish the necessity of the precise auxin dosage and transportation in formation of root nodules.

In conclusion, Xiao et al. (2025) have improved our understanding of how auxin gradient is established through a well-orchestrated mechanism of auxin transportation and local synthesis during nodule primordium development. Interestingly, auxin, like other phytohormones, is also transported through plasmodesmata (Tee and Faulkner 2024). It would be intriguing to further elucidate the roles of plasmodesmata-mediated transport of auxin in nodule development. Collectively, the presented study has created a landscape of spatiotemporal auxin gradient determinants of nodule development in the model legume Medicago. This provides a foundation for further studies aiming to understand atmospheric nitrogen fixation in plants through rhizobia, with a goal to reduce the use of nitrogen fertilizers.

## Data Availability

No data were generated or analyzed in this study.
